# Biomechanical Implications of Mandibular Flexion on Implant-Supported Full-Arch Rehabilitations: A Systematic Literature Review

**DOI:** 10.3390/jcm12165302

**Published:** 2023-08-15

**Authors:** Mario Caggiano, Francesco D’Ambrosio, Alfonso Acerra, David Giudice, Francesco Giordano

**Affiliations:** Department of Medicine, Surgery and Dentistry, University of Salerno, Via Allende, Baronissi, 84081 Salerno, Italy; fradambrosio@unisa.it (F.D.); davidgiudice3@hotmail.com (D.G.); frgiordano@unisa.it (F.G.)

**Keywords:** mandibular flexure, mandibular deformation, fixed oral rehabilitation, implant-supported full-arch

## Abstract

Background: Mandibular flexion (MF) is a complex biomechanical phenomenon, which involves a deformation of the mandible, due mainly to the contraction of the masticatory muscles, and it can have numerous clinical effects. The deformation of the lower jaw caused by mandibular flexion is generally very small, and it is often overlooked and considered irrelevant from a clinical point of view by many authors; however, it should be important to remember that median mandibular flexure (MMF) has a multifactorial aetiology. The main aim of the current systematic review is to highlight the different factors that can increase MF in order to help clinicians identify patients to whom they should pay more attention. As a secondary outcome, we wanted to analyse the preventive measures and suitable techniques to be adopted to minimise the negative effects of this phenomenon on oral fixed rehabilitations. Methods: The review, which was carried out in accordance with the “Preferred Reporting Items for Systematic reviews and Meta-Analyses” (PRISMA) flowchart, was recorded in the “International Prospective Register of Systematic Reviews” (PROSPERO). As research questions, “Patient/Population, Intervention, Comparison and Outcomes” (PICO) questions were employed. Using the ROBINS-I technique, the risk of bias in non-randomised clinical studies was evaluated. Results: The initial electronic search identified over 1300 potential articles, of which 54 studies were included in this systematic review. Information regarding the relationship between MF and individual factors, mandibular movements, impression taking, and fixed rehabilitations were obtained. Conclusions: The studies included in this systematic review showed that MF is greater during protrusive movements, in the posterior areas of the lower jaw, and in patients with brachial facial type, greater jaw length; small gonial angle; and less density, length, and bone surface of the symphysis. The biomechanical effects of mandibular flexion on fixed restorations are debated. Prospective clinical and radiological observational studies should be conducted to evaluate the potential short-, medium-, and long-term consequences of MF.

## 1. Introduction

All long bones in the body exhibit a complex biomechanical behaviour known as elastic-deformation under functional load, and the human mandible is no exception [1]. This is mainly related to two different factors: the intricate structure of the bone, which is an elastic, anisotropic, and non-homogeneous tissue, and its anatomical horseshoe form, which is in close contact with the ligaments and muscles of the head and neck, particularly the masticatory ones [1,2,3,4,5]. The contraction of these structures results in pressure and tractional forces on the mandible, changing its shape. Median mandibular flexure (MMF) is the name for this multifactorial condition, which was first identified around 60 years ago. It more frequently happens when the mouth protrudes or opens, and less frequently when the mouth moves laterally [6,7,8]. Further studies have revealed that it also happens during clenching and bruxism, highlighting that mandibular flexion also occurs with muscular activity alone and not necessarily with jaw movements or when the occlusal load is placed on the jaw itself [9,10,11,12].

The bilateral contraction of the lateral or external pterygoid muscles (LPMs) is the primary source of this phenomenon: when the lower heads contract, they pull the condyles and condylar necks medially, forward and down, producing a buccolingual rotation of the mandibular arch [11]. However, measuring the force generated by the contraction of LPMs to ascertain this is quite difficult due to their size and position [13]. In addition to the lateral pterygoid muscles, the mylohyoid, platysma, superior pharyngeal constrictor, and other jaw depressor muscles provide supplemental aid for its generation [11].

In the frontal plane, the distance between the right and left mandibular ramus narrows due to elastic flexion of the mandible, leading to a reduction in the width of the mandibular arch [6,7,8]. For increasing degrees of jaw opening, mandibular arch static amplitude analyses showed a gradual decrease in its medial–lateral diameter [10,14,15]. Furthermore, dynamic investigations have demonstrated an increase during mandibular retraction and a decrease during protrusion movements, owing to muscular contraction without tooth contact [1,9,11].

Hylander et al. recognised four mandibular deformation patterns during mandibular flexion: symphysis flexion, dorso-ventral shear, corporal rotation, and antero-posterior shear [7]. From his research, it appeared that any of the postulated mandibular deformation patterns can provide compressive, tensile, or shear pressures, and that the highest symphyseal tension, which causes bending, was produced by the contraction of the medial component of the LPMs.

In addition to causing an alteration in the shape of the jaw with a reduction in the width of the arch from a few microns to 1 mm, with an average of 0.073 mm, MMF also affects the relative position of the teeth on the mandibular arch, producing lingual tipping [10,11,14,16,17]. The periodontal ligament reduces bone loss around teeth due to mandibular flexion in natural dentition by allowing the physiological movement of teeth [18,19]. According to Frost’s mechanostatic theory, stress/strain levels are maintained within the bone’s physiological adaptation window by avoiding an excessive rise in stress [20,21]. In the case of edentulous jaws restored with implant-supported full-arch prostheses, a rigid structure is created that connects the various implants, forming a single functional unit [22]. By doing this, not only is there no longer the protective effect of the periodontal ligament, but it facilitates the creation of flexural forces that modify and/or increase bone stress around the implants, resulting in resorption [8,23,24]. Mandibular flexion was found to be the main factor contributing to posterior implant failure in mandibular full-arch fixed prostheses with solidarised implants by Miyamoto et al. [25]. In fixed implant restorations, the biomechanical effect of the mandible’s functional flexibility might result in crestal bone loss surrounding the implant head. Moreover, several clinical and experimental studies have demonstrated that mandibular bending can negatively impact the proper fit of fixed and removable prostheses; lead to denture decementation; and cause fracture of porcelain, screws, or implants [6,17,26,27,28]. Once more, the accuracy of the impression can be impacted by the lingual tipping of the teeth that happens when the mouth is opened for impression taking, creating a series of flaws that could result in treatment failure [15].

As the deformation of the lower jaw caused by mandibular flexion is generally very small, it is often overlooked and considered irrelevant from a clinical point of view by many authors, especially taking into account the large size of the mandible in relation to the lateral pterygoid muscle [27]. However, it should be important to remember that MMF has a multifactorial aetiology and that there are many variables that can affect it and cause increasing deformity up to non-negligible levels. These parameters include facial type; mandibular structure; and symphysis characteristics of bone density, length, and surface area [29,30,31,32,33,34].

This is the background to the present review, the main aim of which is to highlight the different factors that can increase mandibular flexure in order to help clinicians identify patients to whom they should pay more attention [35]. As a secondary outcome, we wanted to analyse the preventive measures and suitable techniques to be adopted to minimise the negative effects of this phenomenon on oral fixed rehabilitations. More emphasis was placed on the different types of fixed full-arch implant-supported rehabilitations. This is intended to facilitate the success of dental therapies aimed at preserving the health of periodontal and peri-implant tissues and achieving long-term outcomes.

## 2. Materials and Methods

### 2.1. Study Protocol

The study protocol was developed in compliance with the Preferred Reporting Items for Systematic Reviews and Meta-analyses (PRISMA) statement before the literature search, data extraction, and analysis and was registered on the “International Prospective Register of Systematic Reviews” (PROSPERO ID: CRD42023438105).

The research question was formulated according to the Population, Intervention, control or Comparison, Outcome (PICO) strategy.

The clinical question in the “PICO” format was as follows: Is there a significant difference in mandibular flexion values based on several factors that may influence fixed oral rehabilitations and what are the preventive measures and suitable techniques to be adopted to minimise the negative effects of this phenomenon on fixed oral rehabilitations?

P (Population): subjects with mandibular oral fixed oral rehabilitations;

I (Intervention): mandibular oral fixed rehabilitations;

C (Comparison): subjects without oral fixed rehabilitations;

O (Outcome): factors that can increase mandibular flexure and the preventive measures and suitable techniques to be adopted to minimise the negative effects of this phenomenon on fixed oral rehabilitations.

### 2.2. Search Strategy

Studies published in the English language concerning factors that can influence mandibular flexure and the influence of MF on oral fixed rehabilitations were electronically searched without date restrictions until 1 May 2023.

An electronic search was conducted through several databases: MEDLINE/PubMed, Google Scholar, BioMed Central, and the Cochrane Library databases, by two independent reviewers (D.G. and A.A.).

The search was performed using the following keywords with Boolean operators:

(“deformation’’ OR “flexion” OR “median flexion” OR “flexure” OR “median flexure”)

AND

(“mandibular” OR “mandible”).

The following filters were applied:

- English language on the MEDLINE/PubMed database.

No filters were employed on the BioMed Central database, Google Scholar, and the Cochrane library.

### 2.3. Study Selection and Eligibility Criteria

The inclusion and exclusion criteria that were established before the start of the search and adhered to when selecting studies are shown below.

Inclusion criteria:

- Studies published in the English language.
- In vivo and in vitro studies.
- Studies examining the effects of mandibular flexion on fixed rehabilitations and the factors influencing it.
- Studies highlighting suitable clinical techniques to be adopted to minimise the negative effects of mandibular flexion.

Exclusion criteria:

- Studies not published in the English language.
- Reviews, systematic reviews, and case reports.
- Studies about the mandibular flexure along with any other physiological or pathological problems.
- Articles that review removable prosthodontic treatments.

Collected citations were recorded, duplicates were eliminated through the Zootero reference manager tool, and the remaining titles were screened by two independent reviewers (D.G. and M.C.). The same two reviewers subsequently screened relevant abstracts of obtained studies.

Full texts of those potentially eligible abstracts were obtained, and full texts were independently reviewed by the same authors (D.G. and F.D.A.). Any disagreement was resolved by discussion, and a third author (A.A.) was consulted in the case of doubt.

The bibliography of the selected articles was examined for relevant titles, and the subsequent study screening was performed as already described.

No restrictions regarding the date of publication or number of studies were applied.

### 2.4. Data Extraction and Collection

Data were independently extracted in duplicate by two authors (D.G. and F.D.A.) on a standardised data extraction form developed from the models proposed for intervention reviews on RCTs and non-RCTs before data extraction; a third author (A.A.) was involved in any case of disagreement. From each study included in the present umbrella review, the following data criteria were recorded:

- Author(s), year and journal of publication, and kind of study;
- Type of rehabilitation, and sample size;
- Factors that can increase mandibular flexure;
- Preventive measures and suitable techniques to be adopted to minimise the negative effects of this phenomenon on oral rehabilitations.

### 2.5. Data Synthesis

The characteristics and main findings of the included studies are presented in tabular form and summarised through a narrative synthesis.

Data from the included studies were qualitatively synthesised through descriptive statistical analysis using the Microsoft Excel software 2019 (Microsoft Corporation, Redmond, WA, USA).

The main aim of this was to highlight the different factors that can increase mandibular flexure, in order to help clinicians identify patients to whom they should pay more attention. As a secondary outcome, we wanted to analyse the preventive measures and suitable techniques to be adopted to minimise the negative effects of this phenomenon on oral rehabilitations.

### 2.6. Quality Assessment

The risk of bias of the non-randomised clinical trials was highlighted by the ROBINS-I (“Risk of Bias In Non-randomised Studies of Interventions”) tool (Sterne, J.A. et al., 2016) [36].

In this tool, different biases are underlined: biases due to confounding, biases due to selection of participants, biases due to classification of interventions, biases due to deviations from intended interventions, biases due to missing data, biases in measurement of outcomes, and biases due to the selection of the reported result (Sterne, J.A. et al., 2016) [36].

The risk of bias assessment was categorised into four levels:

- Low risk of bias: the study is judged to be at low risk of bias for all domains.
- Moderate risk of bias: the study is judged to be at low or moderate risk of bias for all domains.
- Serious risk of bias: the study is judged to be at serious risk of bias in at least one domain, but not at critical risk of bias in any domain.
- Critical risk of bias: the study is judged to be at critical risk of bias in at least one domain.

The response options for the bias are as follows: yes (Y), probably yes (PY), probably no (PN), no (N), and no information (NI). “Y” indicates low risk of bias, “PY” indicates a moderate risk of bias, “PN” indicates a serious risk, “N” indicates a critical risk of bias, and “NI” indicates that there was no information.

## 3. Results

### 3.1. Study Selection

The initial electronic search identified over 1300 potential articles. In particular, 991 records were found using MEDLINE/PubMed, 56 records were found using Google Scholar, 185 records were found using BioMed Central, and 156 records were found using Cochrane Library databases. By manual search, a further 23 articles were identified. Once duplicates were removed, of the 1141 title abstracts identified, only 71 title abstracts were screened. Of these 71 title abstracts, only 61 abstracts were useful for the present systematic review, in accordance with the inclusion criteria. Of these 61 records, their full texts were obtained and screened, and 7 articles were excluded, as shown in Table 1. 

**Figure 1:**
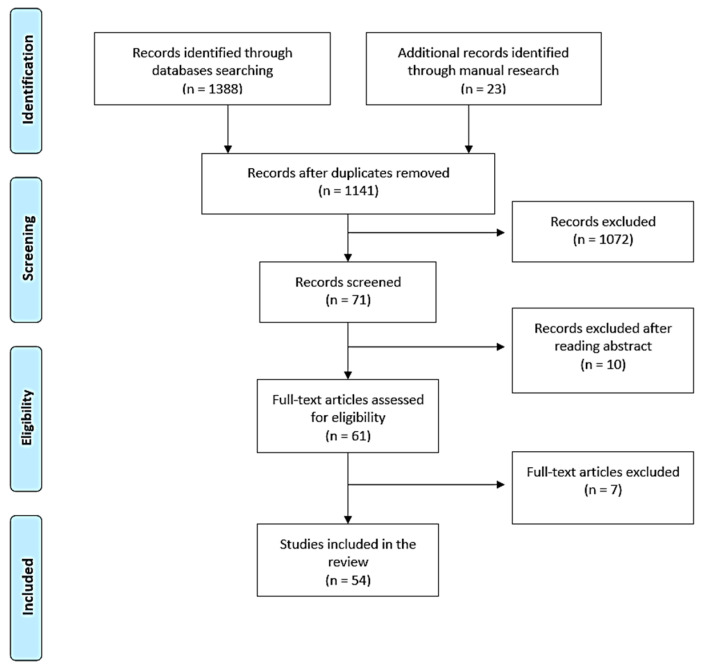
PRISMA flowchart depicting the article selection process.

The table includes author names, years of publication of the studies, the references, and the motivations for the exclusion of the studies from the present review. A total of 54 studies met the eligibility criteria and were included in the review (Figure 1, Table 2).

### 3.2. Data Extraction and Synthesis

Detailed findings related the presence or absence of a significant correlation between mandibular flexion (MF) and various individual factors, as well as the values of MMF according to different types of mandibular movements, are synthesised in Table 3 and Table 4, respectively. The tables also include information about the author(s), the year of publication, and the reference of the articles where these topics are discussed, as well as the type of rehabilitation and the sample size.

The articles concerning the different frameworks of implant-supported full-arch rehabilitations are summarised in Table 5. The table presents information about the author, year of publication and reference of the articles focused on the topic, type of rehabilitation, sample size, and results in favour of divided (D) or undivided (U) frameworks.

From the articles reviewed and summarised in Table 3, it emerges that facial type, gonial angle, length of the mandibular structure, and symphysis characteristics are important individual factors to analyse and take into consideration because they influence the values of mandibular flexion. Instead, it was discovered that the MOF and the variables that affect it had no bearing on mandibular flexion. Regarding gender and age, the results are discordant, and therefore further studies should be conducted.

According to the studies analysed and summarised in Table 4, mandibular flexion values are greater during protrusive movements, mouth opening, and lateral motions in both natural dentition and mandibles rehabilitated with implants.

Table 5 demonstrates the complete lack of consensus in the findings, which prevents us from providing specific recommendations regarding whether to divide the superstructure. This has to do with the fact that there are drawbacks to both single and segmented structures, which will be discussed in the relevant paragraph (type of prosthesis: single or segmented structure).

### 3.3. Quality Assessment of the Included Studies

The evaluation of the risk of bias of the studies included in this systematic review was conducted using the “Risk of Bias in Non-randomised Studies—of Interventions” tool (ROBINS-I), which identifies various sources of bias that can potentially affect the validity and reliability of study findings (Table 6).

## 4. Discussion

All the studies included in the present review established that mandibular bending exists, and most of them focused on calculations and measurements of mandibular deformation during various jaw movements.

### 4.1. Measurement of Mandibular Flexion

Due to the wide variability in jaw size and bone density between individuals, the assessment of mandibular biomechanical characteristics is challenging. In addition, the contraction of the masticatory muscles can generate a wide range of mandibular movements and forces that play a key role in the genesis of MMF. It is extremely difficult to measure the force that the superficial muscles of mastication, such as the masseters, exert on the mandible, and even more so regarding the deep muscles, such as the lateral pterygoid muscles, due to their position and size.

The range of mandibular flexure measurements is a few micrometres to around 1 mm, with an average value of 0.073 mm [11,12,14,16,17,40,44,66,71,77,78].

Such a large range can be justified by several factors affecting the measurements:

- Individual factors: facial type, mandibular structure, gonial angle, and symphysis characteristics (density, length, and bone surface). Some authors have also proposed age, gender, maximum occlusal force (MOF), height, weight, BMI, muscle pain, bruxism, and tooth wear as parameters that may influence mandibular flexion values.
- Measurement techniques: in vivo or in vitro.
- Type of movement performed during measurement: protrusion, mouth opening, laterality, and retrusion.
- Area of the mandible where the measurement is performed: incisor-canine, premolar, and molar area.
- Clinical condition of the mandible: jaw with teeth or edentulous.

#### 4.1.1. Individual Factors

There are three different patterns of facial types:

- Brachifacial is characterised by a reduced angle of the mandibular plane, reduced vertical facial height, and a horizontal growth pattern, with maximum muscle anchorage. Brachifacial patients present a short and wide face, a square jaw and strong muscle chains.
- Mesofacial is characterised by a medium mandibular plane angle, medium vertical facial height, and a mixed growth pattern, with medium muscle anchorage. Mesofacial patients are referred to as “neutral subjects” because no skeletal or muscular features prevail in them, showing a harmonious balance of the vertical and horizontal components of the face.
- Dolichofacial is characterised by a high mandibular plane angle, high vertical facial height, and a vertical growth pattern, with minimal muscle anchorage. Dolichofacial patients have a long, narrow face with a convex profile [79].

From an epidemiological point of view, 70% of the population is mesofacial, while the remaining 30% is divided more or less evenly between brachifacial and dolichofacial types [80]. Since the brachifacial patient has stronger masticatory muscles, it has been hypothesised that they have a higher MMF, followed by the mesofacial and dolichofacial types. This hypothesis has been supported by numerous studies relating facial type to mandibular flexion, all of which were initially conducted on natural dentition [23,26,29,30,34,81]. Nevertheless, Shinkai et al. ruled out a significant influence of facial type on MMF, arguing that, given the small size and not excessive strength of the lateral pterygoid muscle, muscular strength plays a secondary role with respect to the resistance of the bone structure to mandibular deformation [33]. The recent study by Gao, J. et al. evaluated for the first time the morphological-functional response to mandibular flexion of implant-supported prostheses in different facial types, showing that not only is mandibular deformation greater in brachial patients, but that different clinical arrangements are required than in meso and dolicho patients [76].

Mandibular flexion is directly correlated with the length of the mandibular structure: the longer the mandible, the greater the mandibular flexion. Furthermore, it has been shown that the gonial angle, which represents mandibular inclination, when reduced, statistically affects the increase in mandibular flexion, even if to a limited extent [12].

Parameters of considerable influence on MMF are the symphysis characteristics, such as height and length, surface area, and bone density. Several in vivo studies have shown that symphyses with increased length and height, large surface area, and high bone density are more resistant to mandibular deformation, reducing it [7,17,26,30].

On the other hand, older edentulous individuals are more inclined to experience higher mandibular deformation because they have less thick skeletons due to an increased risk of osteoporosis and smaller symphyses as a result of bone resorption after edentulousness [24,44,47,48,50]. However, no age difference was identified in the MMF evaluation between the two groups in the research by Gülsoy et al., where the average age of the edentulous individuals was 63 years and that of the dentate participants was 29 years (*p* > 0.05) [75]. Even though the age–mandibular flexion correlation has not been proven in numerous articles with statistically significant results, the studies showing its interdependence can be explained by the connection between age-related consequences (lower bone density and smaller symphysis structure) and mandibular deformation [73].

Regarding the relationship between sex and mandibular flexion, multiple studies have found that women exhibit more mandibular flexion than males, albeit this difference is not statistically significant [12,30,34,66,77]. A predictability of 32–95.6% was discovered after several research investigated the potential forecasting of sex from mandibular flexion ranges and different mandibular characteristics. The predictive accuracy of mandibular flexion for sex determination was found to be smaller in women than in males by Balci et al. and Shinkai et al. [33,56]—this is probably because women exhibit a greater variability in mandibular arch width. Kemkes-Grottenthaler et al. claim that measurements of the gonial angle, mandibular flexion, and length and width of the ramus can be precise and predictable criteria for morphological identification [52]. However, age and edentulousness may substantially diminish these markers’ accuracy. In the study by Gülsoy et. al., in which no statistically significant difference was found between the sexes in terms of MMF values in both edentulous and dentate patients, the mean MMF values were slightly higher in edentulous males [75]. This is probably due to women experience three times the amount of bone resorption in postmenopausal than men do, and their trabecular bone mineral density decreases more dramatically than men’s does [61].

According to the evaluations of Canabarro and Shinkai et al., R. S. Shinkai et al., and Chen et al., there was no statistically significant correlation between MMF and other parameters such as maximum occlusal force (MOF), height, weight, BMI, muscle pain, bruxism, and tooth wear. It is highly probable that MOF is unrelated to jaw flexion since anthropometric factors (height, weight, and BMI), muscular soreness, bruxism, and tooth wear have a direct proportionality connection with MOF but do not affect mandibular flexion. However, more research is required.

#### 4.1.2. Measurement Techniques

In vitro and in vivo intra- and extra-oral measuring techniques were utilised in the various investigations to analyse the degree of mandibular deformation.

Diagnostic models made from imprints obtained at various phases of the mandibular opening were frequently used to make in vivo extra-oral measurements, as were photos that monitored the movement of the mandibles [10,15,30,73].

On the other hand, strain gauges, calipers, and transducers connected to surfaces or implants were used to make in vivo intra-oral measurements [9,10,14,40,44,77,82].

In vitro measurements to assess the distribution of deformations in the mandibular body were conducted initially using photoelastic models and subsequently by means of FEA, i.e., finite element analysis, which simulates three-dimensional models [75,83,84,85].

However, each of these measuring techniques has disadvantages and limitations. For instance, the strain gauge method can only be used to measure exact strain values at the locations where the strain gauge is placed, whereas the use of photoelastic models has significant numerical data limitations but provides excellent qualitative data on the distribution and concentration of stresses [86]. On the other hand, the finite element method (FEA) can provide detailed quantitative data at any point in the mathematical model. However, in order to obtain accurate results, the modelling must be performed accurately and must look like the real structure [87]. Moreover, modelling biological tissues has several of drawbacks and difficulties, and bone structure can vary both within and between people [75,88]. Compared to models created by scanning traditional impressions, digital models created by intra-oral scanning have been shown to have higher dimensional accuracy: this is mainly related to the fact that, with oral scanning, errors that can arise from an incorrect water/dust ratio of the plaster material and deformation of the impression material are eliminated [89,90,91]. Furthermore, the pressure used to press down on the jaw when obtaining an imprint using conventional techniques may affect mandibular flexibility [71].

#### 4.1.3. Type of Movement Performed during Measurement

**Figure 2:**
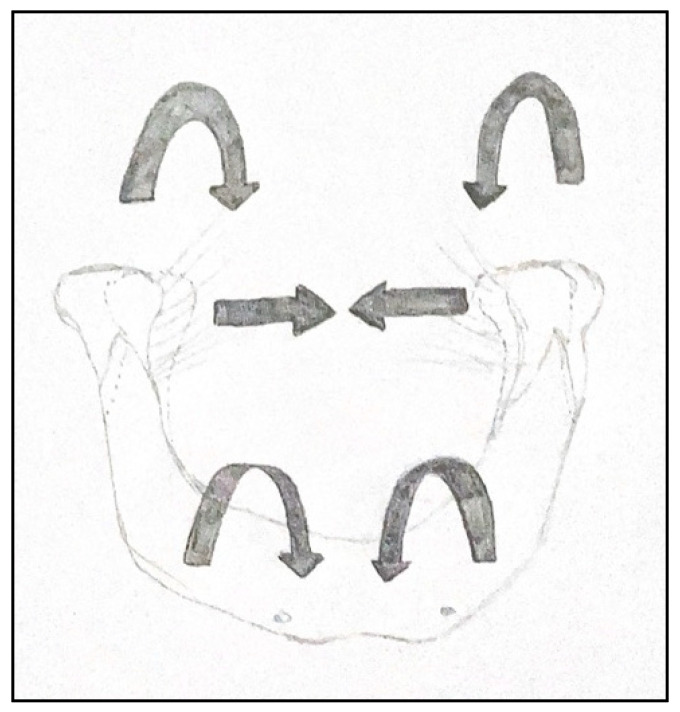
The effects of the contraction of the lateral pterygoid muscles (LPMs) and the consequent mandibular flexion [30].

According to Omar and Wise, there is no change in the mandibular arch width up to a mouth opening of 28% [11]; however, after that point, the decrease is proportionate to the degree of mouth opening, with an average loss of 0.093 mm and a range of 0.012–0.164 mm. The results from this study are comparable to those obtained in the research of Goodkind and Heringlake, and Regli and Kelly, where the deformation ranged from 0.0316 mm to 0.0768 mm and 0.03 mm to 0.09 mm, respectively, depending on the degree of mouth opening [9,10]. Fischman et al. also obtained similar values of mandibular bending, i.e., 0.0711 mm at mouth opening [15]. The values obtained by Chen et al. (2000) on a larger sample (62 volunteers as opposed to Fishman’s 10) are, on the other hand, slightly larger, at 0.145 mm MMF (Figure 2) [30].

Gates and Nicholls demonstrated that mandibular flexion was greater during protrusion movements than during mouth opening movements. In their work, the distortion values found during opening ranged from 0 to 0.3 mm, in line with the studies of Osborne et al., Bowman et al., and Goodkind and Heringlake, but lower than the ranges of 0.2–1.4 mm and 0.6–1.5 mm found by McDowell and Regli, and De Marco and Paine, respectively [6,9,14,42,43]. Conversely, strain values during protrusion range from 0.1 to 0.5 mm, in line with the results obtained by Osborne et al., but lower than the 0.2–1.2 and 0.2–1.5 mm ranges of Bowman et al. and of McDowell and Regli, respectively. Several other clinical and biomechanical studies highlighted the increased mandibular deformation and stress/strain during protrusion movements [58,59,60,69,72]. The lack of involvement of the anterior digastric muscles in mandibular flexion during mouth opening may be the cause of this. From a therapeutic perspective, parafunctions such as grinding or incisal–incisal margin contact can greatly be influenced by this, while for mastication, where protrusive motions are uncommon, it is less significant.

As demonstrated by Burch and Borchers, lateral movements can also cause mandibular arch decrease [44]. In the right lateral position, the average amplitude of the reduction was 0.243 mm, and in the left lateral position, it was 0.257 mm. Due to the activation of only one lateral pterygoid muscle rather than both, the mandibular flexion values in lateral motions are lower than protrusion motions (0.61 mm MMF) and than mouth opening (0.438 mm MMF). The same author then conducted research with a larger sample size (25 participants as opposed to 10 in the prior study), and the same results were validated [1].

Lastly, during retrusion motions, the mandibular arch increases [1,32,51,71,72].

#### 4.1.4. Area of the Mandible Where the Measurement Is Performed

Asadzadeh et al.’s study was the first to examine the potential for various mandibular deformation levels across different mandibular regions [77]. Prior to this investigation, mandibular bending was usually measured at the level of the first or second molar in the posterior intermolar areas. On 35 female volunteers with teeth, Asadzadeh et al. measured MMF using digital calipers in the canines and second molars. In the molar area (0.1894 mm), the mandibular flexure measured greater values than in the canine region (0.1671 mm). This can be explained by the closer proximity of the posterior sectors to the LPM muscle insertions; as one moves toward the anterior sectors from them, mandibular flexion reduces more and more. The recent study by Gülsoy, Tuna, and Pekkan confirmed this hypothesis by taking measurements in seven different regions, starting from the anterior to the posterior region, in dentate and edentulous individuals [75]. To standardise the landmarks in the edentulous individuals, the markers of the dentate persons were employed. In both the toothed and the edentulous specimens, it was shown that the symphyseal region serves as the centre of rotation and that the degree of deformation increases linearly from anterior to posterior locations. The measurements taken on the dentate specimen were 0.048 mm, 0.138 mm, 0.224 mm, 0.324 mm, 0.391 mm, 0.470 mm, and 0.630 mm, whereas the measurements taken on the edentulous specimen were 0.089 mm, 0.162 mm, 0.239 mm, 0.343 mm, 0.452 mm, 0.552 mm, and 0.710 mm. The differences in the MMF values in the molar region of this study compared to past studies can be explained by considering individual factors in the sample and variations related to measurement techniques.

Last but not least, a study by Angel Alvarez Arena revealed that during mouth opening, flexion was greatest at the level of the condyles, slightly less at the level of the body of the mandible, and virtually non-existent in the area of the symphysis, whereas during protrusion, flexion was greatest at the level of the angle of the mandible [32].

#### 4.1.5. Clinical Condition of the Mandible

Following tooth loss, which frequently is brought on by aging, alveolar bone resorbs, and the mineral content and density of cortical and trabecular bone decrease [92]. Mandibular flexion is typically enhanced in low-bone-density patients. However, due to a loss in collagen fibres with age, bone tissue’s elasticity also declines [93]. Considering all of these factors, it follows that mandibular flexion is not significantly different in dentate and edentulous people, nor is it different with age. The study by Gülsoy, Tuna, and Pekkan found no statistically significant difference in the MMF values of the same mandibular areas in dentate and edentulous patients [75].

#### 4.1.6. Potential Recoil of a Mandibular Flexion with a Release of Muscular Tension

A potential recoil of mandibular flexion, accompanied by a release of muscular tension, could lead to several significant effects on the jaw and surrounding structures. As the mandible returns to its original position following flexion, the sudden release of muscular tension may result in a quick and forceful movement. This recoil could potentially cause discomfort or even pain in the temporomandibular joint (TMJ) and surrounding muscles, particularly if the flexion was excessive or performed repetitively. Additionally, abrupt muscular contractions and releases could contribute to increased wear and tear on the teeth, potentially leading to dental issues over time. Careful control and awareness of jaw movements during mandibular flexion are essential to minimise the risk of any adverse effects and maintain optimal oral health [75].

### 4.2. Clinical Effects of MMF

This review revealed a very wide range of mandibular flexion values, from a few micrometres to about 1 mm, with an average of 0.073 mm. Since it is often relatively little, it is frequently disregarded and viewed as useless from a therapeutic perspective. However, more so in protrusive movements and to a lesser extent in mouth-opening movements, in the posterior areas of the mandible and in the presence of individual factors, such as brachi-facial type; long mandibular structure; small gonial angle; and lower symphysis bone density, length, and surface area, the results of the numerous reviewed articles consistently show higher MMF values, which cannot be clinically neglected to preserve periodontal and peri-implant tissue health and achieve long-term outcomes [6,7,12,17,26,30,75,76,77]. Mandibular flexure may affect the precision of the many processes of various prosthetic treatments, especially fixed ones, which might result in failure. It can result in peri-implant bone resorption, distortion of the impression, improper fit of removable or fixed prostheses, fracture of implant screws or porcelain prosthetic crowns, and chewing pain by altering the distribution of masticatory stresses and increasing their intensity in implant-supported prostheses, abutments, and surrounding bone [94]. Consequently, it is essential to implement therapeutic modifications and preventive measures to reduce the negative effects of this phenomenon on oral rehabilitations [23,27,95,96,97,98,99,100,101,102,103].

#### 4.2.1. MMF and Impression Taking

This review clearly showed that, during mouth opening movements, mandibular flexion results in a reduction of the mandibular arch and a lingual tipping of the teeth. All impression taking methods include a certain amount of mouth opening; hence, it is inevitable that the effects of MMF be taken into consideration while creating an impression. Generally, the imperfect fit of dentures was attributed to the variability of dental procedures, not considering the influence of MMF, which can alter the precision of the master model and compromise the prosthesis [9,15]. The prosthesis created from the impression taken with the mouth open wide may not fit the jaw precisely when it is at rest because it is built on a limited arch and has teeth that are not only more lingual but also rotated lingually. This may lead to pressure on the teeth and surrounding structures, pain, gingival inflammation, tooth mobility, and bone loss. The areas generally subject to most pain are located below the lower denture, at the level of the mylohyoid ridge, where the greatest stress occurs during mandibular flexion [40,45]. In implant-supported full-arch prostheses, it is even more important that impressions are accurate to allow a passive fit of the superstructure on rigidly connected implants [23,104]. Consequently, in order to minimise deformation when taking traditional impressions for the lower jaw, it has been suggested that impressions should be made with a minimum mouth opening, as close to the upper jaw as possible and ideally with no more than 20 mm, so as to involve minimal activation of the masticatory muscles [15]. In addition, any protrusive movement should be avoided and, while hardening the impression, the dentist should avoid touching the patient’s jaw, pushing it up or down [41]. The extent of minimisation is uncertain, however, given the limited literature on the subject. For impressions intended for fixed prosthetic rehabilitations, the use of vinyl polyvinyl siloxane (PVS), which has greater dimensional stability than other impression materials, and the use of individual impression posts should be preferred.

When skilled dentists took digital scans instead of traditional impressions, the results were superior, and there was less mandibular bending in the digital scans [17,105].

#### 4.2.2. MMF and Fixed-Teeth-Supported Rehabilitation

By allowing physiological movement of the dental elements, the periodontal ligament (PDL) absorbs most of the stress created by mandibular flexion, preventing bone loss around them [18,19]. However, in fixed-teeth-supported rehabilitations, the use of rigid connectors and long spans limits the movement of the dental components and, as a result, increases stress at the PDL level, which may outcome in bone resorption, as well as at the level of the prosthesis itself, which may end up in porcelain fractures. It is preferable to utilise flexible connections and divide the span into many portions to prevent such unfavourable effects, especially in the case of periodontal patients. Additionally, it is not advised to utilise porcelain for bigger restorations [10,15,46,53].

#### 4.2.3. MMF and Implant-Supported Full-Arch Fixed Rehabilitations

By changing the distribution of stresses at the bone/implant interface and at the level of the prosthetic structure itself, mandibular flexure has the potential to affect the accuracy of several phases of implant rehabilitations, including osseointegration and the creation of implant-supported prostheses. This can result in peri-implant bone resorption, material fracture, and pain during function.

The main goal of implant-supported fixed restorations is to determine an adequate biomechanical distribution both at the level of the prosthetic superstructure and at the level of the implant [69].

To achieve this, it is necessary to make assessments on three different parameters:

- Type of prosthesis: single or segmented structure.
- Material of the superstructure.
- Number and position of implants.

##### Type of Prosthesis: Single or Segmented Structure

The results that have emerged from the literature are somewhat contradictory regarding the necessity or not of splitting the superstructure, separating doctors into two separate schools of thinking. For some authors, division of the superstructure at the level of the symphysis is recommended to reduce the increased stresses occurring at that level [54,62,64]. This indication was also supported by Fischman and McCartney, who highlighted how a single, continuous, and rigid structure can subject both the implant/bone interface and the prosthetic structure to dangerous concentrations of stress, increasing the rate of screw loosening and fracture [15,103]. Other studies favour the undivided superstructure because it can evenly distribute stresses between the splinted implants and its inherent rigidity can provide additional resistance to mandibular bending [57,69]. In any case, all studies agree that it is preferable to divide the structure into two segments at the level of the symphysis rather than three or more segments. Finally, a study by Gao et al. related facial type to prosthetic superstructure [76]. It was found that a one-piece prosthesis is preferable for the brachifacial type, whereas in the case of the mesofacial or dolichofacial type, there is more freedom of choice, with the single structure being preferred if the patient’s other individual factors are correlated with bigger mandibular flexion values.

##### Material of the Superstructure

The material of the superstructure could also influence mandibular bending. Suedam et al. found that materials with a lower modulus of elasticity, and thus that are more flexible, reduce stress to a greater extent, while stiffer materials are more resistant to bending forces [100]. Consequently, they recommended a high modulus of elasticity for the superstructure. This indication was later confirmed by the subsequent work of Favot et al. and rejected by that of Marin et al. [31,39].

Favot described that the zirconia framework has the highest stresses compared with the NiTi. The highest stresses in the framework were obtained during maximum intercuspation. The highest stresses at the bone–implant interface were recorded on the working axial implant during unilateral molar clench and on tilted implants during maximum intercuspation. The influence of the framework’s material stiffness on the stresses at the bone–implant interface was insignificant for axial implants (except the right implant during unilateral molar clench) and slightly more significant for tilted implants. Mandibular flexion decreased with an increase in the cortical bone thickness and the stiffness of the prosthetic framework’s material [31,101,102,103].

Other studies, however, stated that the stiffness of the material used for the prosthesis does not affect mandibular flexion as much as other parameters [47,64,104].

##### Number and Position of Implants

Over the years, several protocols have been proposed for implant-supported fixed rehabilitation of mandibular totally edentulous patients. Brånemark’s initial technique for the rehabilitation of totally edentulous patients involved the use of five implants for the mandible and six for the maxilla arranged in parallel and distributed in the inter-foraminal region for anatomical and surgical reasons, such as the location of the alveolar nerve and the quantity and quality of bone [105,106,107,108,109]. In agreement with the aim of modern dentistry, which is to develop minimally invasive rehabilitations that guarantee functionality, aesthetics, comfort, and cost containment, Malò and Rangert introduced the All-on-4 technique in 2003 as an alternative to much more expensive and invasive methods for the rehabilitation of patients with severe posterior bone atrophy. This technique consisted of the insertion of two axial implants in the lateral incisor/canine area and two implants just mesial to the chin foramen inclined distally at about 30° to the occlusal plane with the implant plate near the second premolar and prosthetic superstructure with distal cantilevers [110]. However, several clinical and virtual studies, using finite-element analysis (FEA), have shown that restorations with distal cantilevers can lead to detrimental biomechanical stress on the peri-implant bone, due to unfavourable lever arms [110,111,112,113]. Shackleton et al., White et al., Lindquist et al., and Naert et al. confirmed the potential negative effects of distal cantilevers, showing a lower success rate and peri-implant bone loss [98,114,115,116]. Fenton and Zarb interpreted these results, suggesting the placement of multiple implants to achieve an even distribution of stresses and avoid long cantilevers [117]. On this line of thought, Agliardi et al. proposed the All-on-6 technique [118].

To date, there is no uniformity of thought on either the precise number of implants or their positioning in the arch for ideal rehabilitation. Over the years, several studies have been conducted to evaluate the effects of mandibular flexion on individual implants in different types of rehabilitation. From the reviewed articles, a controversy has arisen regarding the placement of implants mesial or distal to the chin foramen: some scholars, including Zarone et al., prefer their placement more mesial, allowing for less pressure on the implants; others, including Nokar et al., recommend placing implants also more distal, demonstrating that peri-implant bone stress is less in this position [54,64]. The lack of unanimity of results may be due to the large number of parameters influencing mandibular flexion in implant-supported restorations.

The biomechanical effects of mandibular flexion on fixed-implant-supported restorations are debated. Considering the inhomogeneity of results with regard to the number and position of implants, as well as the segmentation or non-segmentation of the superstructure and its material of construction, further prospective clinical and radiological observational studies should be conducted to evaluate the potential short-, medium-, and long-term consequences of mandibular flexure on implant-supported full-arch restorations.

## 5. Conclusions

Mandibular flexion is greater during protrusive movements, in the posterior areas of the lower jaw, and in patients with brachial facial type; greater jaw length; small gonial angle; and less density, length, and bone surface of the symphysis. The various articles reviewed showed no statistically significant difference in the extent of mandibular flexion between the right and left hemiarch; between edentulous and toothless patients; and when parameters such as age, gender, maximum occlusal force (MOF), height, weight, BMI, muscle pain, bruxism, and tooth wear were varied.

This review revealed a very wide range of mandibular flexion values, from a few micrometres to approximately 1 mm, with a mean of 0.073 mm, so given the negligible values, this is often ignored and seen as useless from a therapeutic perspective.

Nonetheless, to minimise its negative effect and achieve long-term outcomes, certain preventive measures and suitable techniques must be adopted during the different phases of oral rehabilitations.

The literature suggests that the impression of the lower jaw should be taken with a minimum mouth opening, as close to the upper jaw as possible and ideally no more than 20 mm, to involve minimal activation of the masticatory muscles. In addition, any protrusive movement should be avoided and, while hardening the impression, the dentist should avoid touching the patient’s jaw by pushing it up or down. Finally, digital scans showed minimal mandibular flexion and more effective results than traditional impressions.

Regarding fixed-tooth-supported rehabilitations, it is advisable to use non-rigid connectors and to divide the span into several sections, even more so in the case of periodontal patients. Furthermore, the use of porcelain in larger restorations is not recommended.

## Figures and Tables

**Table 1:** Studies excluded along with reasons for exclusion.

Author, Year of Publication and Reference	Reason for Exclusion
Van Eijden, T.M., 2000 [8]	It is a review
Paez, C.Y., 2003 [28]	It is a case report
de Oliveira, R.M., 2000 [37]	It is a case report
Law, C., 2012 [38]	It is a review
Marin, D.O., 2015 [39]	It is a case report
Sivaraman, K., 2016 [40]	It is a review
Mijiritsky, E., 2022 [41]	It is a narrative review

**Table 2:** Characteristics of the studies included in the present systematic review: author, year of publication, and reference; journal of publication; study design; outcome. MF: mandibular flexion.

Author, Year of Publication and Reference	Journal of Publication	Study Design	Outcome
Burch, J.G., 1972 [1]	*Arch. Oral Biol.*	Clinical trial	Influence of mandibular movements on MF values
Gates, G.N., 1981 [6]	*J. Prosthet. Dent.*	Clinical trial	Influence of mandibular movements on MF values
Hylander, W.L., 1984 [7]	*Am. J. Phys. Anthropol.*	Clinical trial	Influence of individual factors on MF values
Goodkind, R.J., 1973 [9]	*J. Prosthet. Dent.*	Clinical trial	MF measurement
Regli, C.P., 1967 [10]	*J. Prosthet. Dent.*	Clinical trial	MF measurement
Omar, R., 1981 [11]	*J. Oral Rehabil.*	Clinical trial	Influence of MF on impression taking
Canabarro Sde, A., 2006 [12]	*Int. J. Prosthodont.*	Clinical trial	Influence of mandibular movements and individual factors on MF values
De Marco, T.J., 1974 [14]	*J. Prosthet. Dent.*	Clinical trial	MF measurement
Fischman, B., 1990 [15]	*J. Prosthet. Dent.*	Clinical trial	MF measurement
Shinkai, R., 2004 [17]	*J. Appl. Oral Sci.*	Clinical trial	Influence of individual factors on MF values
Hobkirk, J.A., 1998 [23]	*J. Prosthet. Dent.*	Clinical trial	Influence of individual factors on MF values
Hobkirk, J.A., 1991 [26]	*Int. J. Oral Maxillofac. Implant.*	Clinical trial	Influence of individual factors on MF values
Horiuchi, M., 1997 [27]	*Arch. Oral Biol.*	Clinical trial	Influence of mandibular movements on MF values
Custodio, W., 2011 [29]	*J. Appl. Oral Sci.*	Clinical trial	Influence of individual factors on MF values
Chen, D.C., 2000 [30]	*J. Dent.*	Clinical trial	Influence of individual factors on MF values
Favot, L.M., 2014 [31]	*J. Dent.*	Clinical trial	MF values with different superstructure’s material and cortical bone thickness
Alvarez-Arenal, A., 2009 [32]	*Math. Comput. Model.*	Clinical trial	Influence of mandibular movements and individual factors on MF values
Shinkai, R.S., 2007 [33]	*Head Face Med.*	Clinical trial	Influence of individual factors on MF values
Prasad, M., 2013 [34]	*J. Nat. Sci. Biol. Med.*	Clinical trial	Influence of individual factors on MF values
McDowell, J.A., 1961 [42]	*J. Dent. Res.*	Clinical trial	Influence of mandibular movements on MF values
Osborne, J., 1964 [43]	*Br. Dent. J.*	Clinical trial	MF measurement
Burch, J.G., 1970 [44]	*J. Dent. Res.*	Clinical trial	Influence of mandibular movements and individual factors on MF values
Novak, C.A., 1972 [45]	*Dent. Stud.*	Clinical trial	MF measurement
Fischman, B.M., 1976 [46]	*J. Prosthet. Dent.*	Clinical trial	MF reduces when fixed splints are present in natural dentition
Ferrario, V., 1992 [47]	*J. Prosthet. Dent.*	Clinical trial	Influence of individual factors on MF values
Hart, R.T., 1992 [48]	*J. Biomech.*	Clinical trial	Influence of individual factors on MF values
Korioth, T.W., 1992 [49]	*Am. J. Phys. Anthropol.*	Clinical trial	Influence of individual factors on MF values
Koolstra, J.H., 1995 [50]	*J. Dent. Res.*	Clinical trial	Influence of individual factors on MF values
Abdel-Latif, H.H., 2000 [51]	*Int. J. Prosthodont.*	Clinical trial	MF measurement
Kemkes-Grottenthaler, A., 2002 [52]	*Homo*	Clinical trial	Influence of individual factors on MF values
Jiang, T., 2002 [53]	*J. Oral Rehabil.*	Clinical trial	Influence of MF on connected prosthesis supported by natural tooth and implants
Zarone, F., 2003 [54]	*Clin. Oral Implant. Res.*	Clinical trial	Influence of MF on implants and superstructures in different fixed full-arch rehabilitations
Choi, A.H., 2005 [55]	*Aust. Dent. J.*	Clinical trial	Influence of mandibular movements on MF values
Balci, Y., 2005 [56]	*Homo*	Clinical trial	Influence of individual factors on MF values
Yokoyama, S., 2005 [57]	*Int. J. Oral Maxillofac. Implant.*	Clinical trial	Influence of MF on different superstructures in fixed full-arch rehabilitations
Al-Sukhun, J., 2006 [58]	*J. Oral Maxillofac. Surg.*	Clinical trial	Influence of mandibular movements on MF values
Al-Sukhun, J., 2007 [59]	*Int. J. Oral Maxillofac. Implant.*	Clinical trial	Influence of mandibular movements on MF values
El-Sheikh, A.M., 2007 [60]	*Int. J. Oral Maxillofac. Implant.*	Clinical trial	Influence of mandibular movements on MF values
Gulsahi, A., 2008 [61]	*Dentomaxillofac. Radiol.*	Clinical trial	Influence of individual factors on MF values
Naini, R.B., 2009 [62]	*Implant. Dent.*	Clinical trial	Influence of MF on different superstructures in fixed full-arch rehabilitations
Bellini, C.M., 2009 [63]	*Int. J. Oral Maxillofac. Implant.*	Clinical trial	Influence of MF on tilted and nontilted implant
Nokar, S., 2010 [64]	*Int. J. Oral Maxillofac. Implant.*	Clinical trial	Influence of MF on different superstructures in fixed full-arch rehabilitations
Zaugg, B., 2012 [65]	*Clin. Oral Implant. Res.*	Clinical trial	MF values in oral rehabilitation with posterior implants and natural teeth in anterior mandible
Madani, A.S., 2012 [66]	*J. Dent. Mater. Tech.*	Clinical trial	Influence of individual factors on MF values
Law, C., 2014 [67]	*J. Prosthet. Dent.*	Clinical trial	Influence of MF on the strain distribution in unilateral distal edentulisms
Lin, C., 2014 [68]	*Forensic Sci. Int.*	Clinical trial	Influence of individual factors on MF values
Martin-Fernandez, E., 2018 [69]	*Biomed. Res. Int.*	Clinical trial	Influence of superstructure type and different mandibular movements on MF in fixed implant rehabilitations
Shahriari, S., 2019 [70]	*J. Long. Term. Eff. Med. Implant.*	Clinical trial	Influence of MF on tilted and nontilted implant
Wolf, L., 2019 [71]	*Int. J. Comput. Dent.*	Clinical trial	Influence of mandibular movements and individual factors on MF values
Tulsani, M., 2020 [72]	*Int. J. Dent. Oral Sci. *	Clinical trial	Influence of mandibular movements on MF values
Ebadian, B., 2020 [73]	*J. Indian Prosthodont. Soc.*	Clinical trial	Influence of individual factors on MF values
Schmidt, A., 2021 [74]	*Clin. Oral Investig.*	Clinical trial	Influence of MF on different techniques of impression taking
Gülsoy, M., 2022 [75]	*J. Adv. Prosthodont.*	Clinical trial	Influence of individual factors on MF values
Gao, J., 2022 [76]	*Front. Bioeng. Biotechnol.*	Clinical trial	Influence of individual factors on MF values

**Table 3:** Summary of studies included in the current review regarding the existence of significant correlation (+) or not (-) between MF (mandibular flexion) and individual factors *. * Age, sex, facial type, gonial angle, length of the mandibular structure, symphysis characteristics, and MOF (maximum occlusal force) and parameters that modify it (height, weight, BMI, muscle pain, bruxism, and tooth wear).

Author, Year of Publication, and Reference	Type of Rehabilitation	Sample Size	Correlation between MF and Individual Factors *
Hylander, W.L., 1984 [7]	Natural dentition	6 macaca fascicularis	Symphysis characteristics +
Canabarro Sde, A., 2006 [12]	Natural dentition	80	Gonial angle + Length of the mandibular structure + Sex - Age - MOF and parameters that modify it -
Shinkai, R., 2004 [17]	Natural dentition	7	Symphysis characteristics +
Hobkirk, J.A., 1998 [23]	Natural dentition	3	Facial type +
Hobkirk, J.A., 1991 [26]	Natural dentition	3	Facial type + Symphysis characteristics +
Custodio, W., 2011 [29]	Natural dentition	78	Facial type +
Chen, D.C., 2000 [30]	Natural dentition	62	Facial type + Gonial angle + Symphysis characteristics + Sex - MOF and parameters that modify it -
Shinkai, R.S., 2007 [33]	Natural dentition	51	Facial type - Sex + MOF and parameters that modify it -
Prasad, M., 2013 [34]	Natural dentition	60	Facial type + Sex -
Burch, J.G., 1970 [44]	Natural dentition	10	Age +
Ferrario, V., 1992 [47]	Natural dentition	3D FEM	Age +
Hart, R.T., 1992 [48]	Natural dentition	3D FEM	Age +
Korioth, T.W., 1992 [49]	Natural dentition	3D FEM	Age +
Koolstra, J.H., 1995 [50]	Natural dentition	3D FEM	Age +
Kemkes-Grottenthaler, A., 2002 [52]	Forensic mandibles and archaeological mandibles	153 forensic mandibles and 80 archaeological mandibles	Sex +
Balci, Y., 2005 [56]	Forensic mandibles	120 mandibles from forensic cases	Sex +
Gulsahi, A., 2008 [61]	Edentulous, partially, and full dentate patients	1.863	Sex +
Madani, A.S., 2012 [66]	Natural dentition and edentulous	50 and 70	Age -
Lin, C., 2014 [68]	Natural dentition	3D FEM	Sex +
Wolf, L., 2019 [71]	Natural dentition	40	Sex -
Ebadian, B., 2020 [73]	Natural dentition	90	Age + Sex - MOF and parameters that modify it -
Gülsoy, M., 2022 [75]	Natural dentition and edentulous	56 and 35	Age - Sex -
Gao, J., 2022 [76]	Implant-supported fixed restorations	3D FEM	Facial type +

**Table 4:** Summary of studies included in the current review regarding the values of MMF according to different types of mandibular movements *. * Mouth opening, protrusion, and lateral movements.

Author, Year of Publication, and Reference	Type of Rehabilitation	Sample Size	Type of Movement * and Values of MMF
Burch, J.G., 1972 [1]	Natural dentition	25	Mouth opening 0.224 mm Protrusion 0.432 mm Lateral movements 0.112/0.105 mm
Gates, G.N., 1981 [6]	Natural dentition	10	Mouth opening 0–0.3 mm Protrusion 0.1–0.5 mm
Goodkind, R.J., 1973 [9]	Natural dentition	40	Mouth opening 0.031–0.076 mm
Regli, C.P., 1967 [10]	Natural dentition	62	Mouth opening 0.03–0.09 mm
Omar, R., 1981 [11]	Natural dentition	10	Mouth opening 0.012–0.164 mm
Canabarro Sde, A., 2006 [12]	Natural dentition	80	Mouth opening 0.146 mm Protrusion 0.15 mm
De Marco, T.J., 1974 [14]	Natural dentition	25	Mouth opening 0.78 mm
Fischman, B., 1990 [15]	Natural dentition	10	Mouth opening 0.0711 mm
Shinkai, R., 2004 [17]	Natural dentition	7	Mouth opening 0.21–0.44 mm
Horiuchi, M., 1997 [27]	Natural dentition	4	Mouth opening 0.016 mm Protrusion 0.010–0.037 mm
Chen, D.C., 2000 [30]	Natural dentition	62	Mouth opening 0.145 mm
McDowell, J.A., 1961 [42]	Natural dentition	20	Mouth opening 0.4 mm Protrusion 0.5 mm
Osborne, J., 1964 [43]	Natural dentition	18	Mouth opening 0.07 mm
Burch, J.G., 1970 [44]	Natural dentition	10	Mouth opening 0.438 mm Protrusion 0.61 mm Lateral movements 0.243/0.257 mm
Novak, C.A., 1972 [45]	Natural dentition	50	Mouth opening 1.00 mm
Choi, A.H., 2005 [55]	Edentulous mandible with implants	3D FEM	Mouth opening 0.168 mm in the first molar region and 0.256 mm in the second molar region
Al-Sukhun, J., 2006 [58]	Edentulous patients with implants	12	Mouth opening 0.011–0.052 mm Protrusion 0.025–0.057 mm
Al-Sukhun, J., 2007 [59]	Edentulous patients with implants	12	Mouth opening 0.8 mm Protrusion 1.07 mm Lateral movements 1.1/0.9 mm
El-Sheikh, A.M., 2007 [60]	Edentulous patients with implants	5	Mouth opening 0.025–0.042 mm Protrusion 0.018–0.053 mm Lateral movements 0.010–0.021 mm
Madani, A.S., 2012 [66]	Natural dentition and edentulous	50 and 70	Mouth opening 0.078–0.751 mm
Wolf, L., 2019 [71]	Natural dentition	40	Mouth opening 0.011–0.232 mm
Tulsani, M., 2020 [72]	Natural dentition	140	Mouth opening 0.363 mm Protrusion 0.973 mm

**Table 5:** Summary of studies included in the current review regarding different types of superstructures of full-arch implant-supported rehabilitation. ^1^ D: division of the superstructure; U: undivided framework.

Author, Year of Publication, and Reference	Type of Rehabilitation	Sample Size	Results in Favour of D/U ^1^
Zarone, F., 2003 [54]	Full-arch 6-implant-supported rehabilitation	1	D
Yokoyama, S., 2005 [57]	Full-arch 8-implant-supported rehabilitation	3D FEM	U
Naini, R.B., 2009 [62]	Full-arch 5-implant-supported rehabilitation	3D FEM	D
Nokar, S., 2010 [64]	Full-arch 6-implant-supported rehabilitation	3D FEM	D
Martin-Fernandez, E., 2018 [69]	Full-arch 6-implant-supported rehabilitation	3D FEM	U
Gao, J., 2022 [76]	Full-arch implant-supported rehabilitation	3D FEM	U

**Table 6:** Risk of bias of the studies included in the systematic review. The response options for the bias are as follows: yes (Y), probably yes (PY), probably no (PN), no (N), and no information (NI). “Y” indicates low risk of bias, “PY” indicates a moderate risk of bias; “PN” indicates a serious risk, “N” indicates a critical risk of bias, and “NI” indicates that there are no data. The value in bold is the response option of the article bias.

Studies	Bias Due to Confounding	Bias in Selection of Participants	Bias in Measurement Classification of Interventions	Bias Due to Deviations from Intended Interventions	Bias Due to Missing Data	Bias in Measurement of Outcomes	Bias Due to Selection of the Reported Result
Burch, J.G., 1972 [1]	Y/**PY**/ PN/N	**Y**/PY/ PN/N/NI	Y/**PY**/ PN/N/NI	**Y**/PY/ PN/N/NI	**Y**/PY/ PN/N/NI	**Y**/PY/ PN/N/NI	**Y**/PY/ PN/N/NI
Gates, G.N., 1981 [6]	Y/**PY**/ PN/N	**Y**/PY/ PN/N/NI	**Y**/PY/ PN/N/NI	**Y**/PY/ PN/N/NI	**Y**/PY/ PN/N/NI	**Y**/PY/ PN/N/NI	**Y**/PY/ PN/N/NI
Hylander, W.L., 1984 [7]	**Y**/PY/ PN/N	**Y**/PY/ PN/N/NI	**Y**/PY/ PN/N/NI	**Y**/PY/ PN/N/NI	**Y**/PY/ PN/N/NI	**Y**/PY/ PN/N/NI	Y/**PY**/ PN/N/NI
Goodkind, R.J., 1973 [9]	Y/**PY**/ PN/N	Y/**PY**/ PN/N/NI	Y/**PY**/ PN/N/NI	**Y**/PY/ PN/N/NI	**Y**/PY/ PN/N/NI	**Y**/PY/ PN/N/NI	Y/**PY**/ PN/N/NI
Regli, C.P., 1967 [10]	**Y**/PY/ PN/N	Y/**PY**/ PN/N/NI	**Y**/PY/ PN/N/NI	**Y**/PY/ PN/N/NI	**Y**/PY/ PN/N/NI	**Y**/PY/ PN/N/NI	**Y**/PY/ PN/N/NI
Omar, R., 1981 [11]	Y/**PY**/ PN/N	**Y**/PY/ PN/N/NI	**Y**/PY/ PN/N/NI	**Y**/PY/ PN/N/NI	**Y**/PY/ PN/N/NI	**Y**/PY/ PN/N/NI	**Y**/PY/ PN/N/NI
Canabarro Sde, A., 2006 [12]	**Y**/PY/ PN/N	**Y**/PY/ PN/N/NI	**Y**/PY/ PN/N/NI	**Y**/PY/ PN/N/NI	**Y**/PY/ PN/N/NI	**Y**/PY/ PN/N/NI	**Y**/PY/ PN/N/NI
De Marco, T.J., 1974 [14]	Y/PY/ **PN**/N	Y/PY/ **PN**/N/NI	**Y**/PY/ PN/N/NI	**Y**/PY/ PN/N/NI	**Y**/PY/ PN/N/NI	**Y**/PY/ PN/N/NI	Y/**PY**/ PN/N/NI
Fischman, B., 1990 [15]	**Y**/PY/ PN/N	**Y**/PY/ PN/N/NI	**Y**/PY/ PN/N/NI	**Y**/PY/ PN/N/NI	Y/**PY**/ PN/N/NI	Y/**PY**/ PN/N/NI	**Y**/PY/ PN/N/NI
Shinkai, R., 2004 [17]	Y/**PY**/ PN/N	**Y**/PY/ PN/N/NI	**Y**/PY/ PN/N/NI	**Y**/PY/ PN/N/NI	**Y**/PY/ PN/N/NI	**Y**/PY/ PN/N/NI	**Y**/PY/ PN/N/NI
Hobkirk, J.A., 1998 [23]	Y/**PY**/ PN/N	**Y**/PY/ PN/N/NI	**Y**/PY/ PN/N/NI	**Y**/PY/ PN/N/NI	**Y**/PY/ PN/N/NI	**Y**/PY/ PN/N/NI	**Y**/PY/ PN/N/NI
Hobkirk, J.A., 1991 [26]	**Y**/PY/ PN/N	Y/**PY**/ PN/N/NI	**Y**/PY/ PN/N/NI	**Y**/PY/ PN/N/NI	**Y**/PY/ PN/N/NI	**Y**/PY/ PN/N/NI	**Y**/PY/ PN/N/NI
Horiuchi, M., 1997 [27]	Y/**PY**/ PN/N	**Y**/PY/ PN/N/NI	**Y**/PY/ PN/N/NI	**Y**/PY/ PN/N/NI	**Y**/PY/ PN/N/NI	**Y**/PY/ PN/N/NI	**Y**/PY/ PN/N/NI
Custodio, W., 2011 [29]	Y/**PY**/ PN/N	**Y**/PY/ PN/N/NI	**Y**/PY/ PN/N/NI	**Y**/PY/ PN/N/NI	**Y**/PY/ PN/N/NI	**Y**/PY/ PN/N/NI	**Y**/PY/ PN/N/NI
Chen, D.C., 2000 [30]	Y/**PY**/ PN/N	**Y**/PY/ PN/N/NI	**Y**/PY/ PN/N/NI	**Y**/PY/ PN/N/NI	**Y**/PY/ PN/N/NI	**Y**/PY/ PN/N/NI	**Y**/PY/ PN/N/NI
Favot, L.M., 2014 [31]	Y/**PY**/ PN/N	**Y**/PY/ PN/N/NI	**Y**/PY/ PN/N/NI	**Y**/PY/ PN/N/NI	**Y**/PY/ PN/N/NI	**Y**/PY/ PN/N/NI	Y/**PY**/ PN/N/NI
Alvarez-Arenal, A., 2009 [32]	Y/**PY**/ PN/N	Y/**PY**/ PN/N/NI	**Y**/PY/ PN/N/NI	**Y**/PY/ PN/N/NI	**Y**/PY/ PN/N/NI	**Y**/PY/ PN/N/NI	**Y**/PY/ PN/N/NI
Shinkai, R.S., 2007 [33]	**Y**/PY/ PN/N	**Y**/PY/ PN/N/NI	**Y**/PY/ PN/N/NI	**Y**/PY/ PN/N/NI	**Y**/PY/ PN/N/NI	**Y**/PY/ PN/N/NI	**Y**/PY/ PN/N/NI
Prasad, M., 2013 [34]	Y/**PY**/ PN/N	**Y**/PY/ PN/N/NI	**Y**/PY/ PN/N/NI	**Y**/PY/ PN/N/NI	**Y**/PY/ PN/N/NI	**Y**/PY/ PN/N/NI	**Y**/PY/ PN/N/NI
McDowell, J.A., 1961 [42]	Y/**PY**/ PN/N	Y/**PY**/ PN/N/NI	Y/**PY**/ PN/N/NI	**Y**/PY/ PN/N/NI	**Y**/PY/ PN/N/NI	**Y**/PY/ PN/N/NI	**Y**/PY/ PN/N/NI
Osborne, J., 1964 [43]	Y/**PY**/ PN/N	Y/**PY**/ PN/N/NI	**Y**/PY/ PN/N/NI	**Y**/PY/ PN/N/NI	**Y**/PY/ PN/N/NI	**Y**/PY/ PN/N/NI	**Y**/PY/ PN/N/NI
Burch, J.G., 1970 [44]	**Y**/PY/ PN/N	**Y**/PY/ PN/N/NI	**Y**/PY/ PN/N/NI	**Y**/PY/ PN/N/NI	**Y**/PY/ PN/N/NI	**Y**/PY/ PN/N/NI	**Y**/PY/ PN/N/NI
Novak, C.A., 1972 [45]	Y/**PY**/ PN/N	**Y**/PY/ PN/N/NI	**Y**/PY/ PN/N/NI	**Y**/PY/ PN/N/NI	**Y**/PY/ PN/N/NI	**Y**/PY/ PN/N/NI	**Y**/PY/ PN/N/NI
Fischman, B.M., 1976 [46]	**Y**/PY/ PN/N	**Y**/PY/ PN/N/NI	**Y**/PY/ PN/N/NI	**Y**/PY/ PN/N/NI	**Y**/**PY**/ PN/N/NI	**Y**/**PY**/ PN/N/NI	**Y**/PY/ PN/N/NI
Ferrario, V., 1992 [47]	Y/**PY**/ PN/N	Y/**PY**/ PN/N/NI	**Y**/PY/ PN/N/NI	**Y**/PY/ PN/N/NI	**Y**/PY/ PN/N/NI	**Y**/PY/ PN/N/NI	Y/**PY**/ PN/N/NI
Hart, R.T., 1992 [48]	Y/**PY**/ PN/N	Y/**PY**/ PN/N/NI	**Y**/PY/ PN/N/NI	**Y**/PY/ PN/N/NI	**Y**/PY/ PN/N/NI	**Y**/PY/ PN/N/NI	**Y**/PY/ PN/N/NI
Korioth, T.W., 1992 [49]	Y/**PY**/ PN/N	**Y**/PY/ PN/N/NI	**Y**/PY/ PN/N/NI	**Y**/PY/ PN/N/NI	**Y**/PY/ PN/N/NI	**Y**/PY/ PN/N/NI	**Y**/PY/ PN/N/NI
Koolstra, J.H., 1995 [50]	Y/**PY**/ PN/N	**Y**/PY/ PN/N/NI	**Y**/PY/ PN/N/NI	**Y**/PY/ PN/N/NI	**Y**/PY/ PN/N/NI	**Y**/PY/ PN/N/NI	**Y**/PY/ PN/N/NI
Abdel-Latif, H.H., 2000 [51]	Y/**PY**/ PN/N	**Y**/PY/ PN/N/NI	**Y**/PY/ PN/N/NI	**Y**/PY/ PN/N/NI	**Y**/PY/ PN/N/NI	**Y**/PY/ PN/N/NI	**Y**/PY/ PN/N/NI
Kemkes-Grottenthaler, A., 2002 [52]	Y/**PY**/ PN/N	**Y**/PY/ PN/N/NI	**Y**/PY/ PN/N/NI	**Y**/PY/ PN/N/NI	**Y**/PY/ PN/N/NI	**Y**/PY/ PN/N/NI	**Y**/PY/ PN/N/NI
Jiang, T., 2002 [53]	Y/**PY**/ PN/N	**Y**/PY/ PN/N/NI	**Y**/PY/ PN/N/NI	**Y**/PY/ PN/N/NI	**Y**/PY/ PN/N/NI	**Y**/PY/ PN/N/NI	Y/**PY**/ PN/N/NI
Zarone, F., 2003 [54]	**Y**/PY/ PN/N	**Y**/PY/ PN/N/NI	**Y**/PY/ PN/N/NI	**Y**/PY/ PN/N/NI	**Y**/PY/ PN/N/NI	**Y**/PY/ PN/N/NI	**Y**/PY/ PN/N/NI
Choi, A.H., 2005 [55]	Y/**PY**/ PN/N	Y/**PY**/ PN/N/NI	**Y**/PY/ PN/N/NI	**Y**/PY/ PN/N/NI	**Y**/PY/ PN/N/NI	**Y**/PY/ PN/N/NI	**Y**/PY/ PN/N/NI
Balci, Y., 2005 [56]	Y/**PY**/ PN/N	**Y**/PY/ PN/N/NI	**Y**/PY/ PN/N/NI	**Y**/PY/ PN/N/NI	**Y**/PY/ PN/N/NI	**Y**/PY/ PN/N/NI	**Y**/PY/ PN/N/NI
Yokoyama, S., 2005 [57]	Y/**PY**/ PN/N	**Y**/PY/ PN/N/NI	**Y**/PY/ PN/N/NI	**Y**/PY/ PN/N/NI	**Y**/PY/ PN/N/NI	**Y**/PY/ PN/N/NI	**Y**/PY/ PN/N/NI
Al-Sukhun, J., 2006 [58]	**Y**/PY/ PN/N	**Y**/PY/ PN/N/NI	**Y**/PY/ PN/N/NI	**Y**/PY/ PN/N/NI	**Y**/PY/ PN/N/NI	**Y**/PY/ PN/N/NI	Y/**PY**/ PN/N/NI
Al-Sukhun, J., 2007 [59]	**Y**/PY/ PN/N	**Y**/PY/ PN/N/NI	**Y**/PY/ PN/N/NI	**Y**/PY/ PN/N/NI	**Y**/PY/ PN/N/NI	**Y**/PY/ PN/N/NI	**Y**/PY/ PN/N/NI
El-Sheikh, A.M., 2007 [60]	**Y**/PY/ PN/N	**Y**/PY/ PN/N/NI	**Y**/PY/ PN/N/NI	**Y**/PY/ PN/N/NI	**Y**/PY/ PN/N/NI	**Y**/PY/ PN/N/NI	**Y**/PY/ PN/N/NI
Gulsahi, A., 2008 [61]	Y/**PY**/ PN/N	**Y**/PY/ PN/N/NI	**Y**/PY/ PN/N/NI	**Y**/PY/ PN/N/NI	**Y**/PY/ PN/N/NI	**Y**/PY/ PN/N/NI	**Y**/PY/ PN/N/NI
Naini, R.B., 2009 [62]	**Y**/PY/ PN/N	**Y**/PY/ PN/N/NI	**Y**/PY/ PN/N/NI	**Y**/PY/ PN/N/NI	**Y**/PY/ PN/N/NI	**Y**/PY/ PN/N/NI	**Y**/PY/ PN/N/NI
Bellini, C.M., 2009 [63]	**Y**/PY/ PN/N	**Y**/PY/ PN/N/NI	**Y**/PY/ PN/N/NI	**Y**/PY/ PN/N/NI	**Y**/PY/ PN/N/NI	**Y**/PY/ PN/N/NI	Y/**PY**/ PN/N/NI
Nokar, S., 2010 [64]	**Y**/PY/ PN/N	Y/**PY**/ PN/N/NI	**Y**/PY/ PN/N/NI	**Y**/PY/ PN/N/NI	**Y**/PY/ PN/N/NI	**Y**/PY/ PN/N/NI	**Y**/PY/ PN/N/NI
Zaugg, B., 2012 [65]	Y/**PY**/ PN/N	**Y**/PY/ PN/N/NI	**Y**/PY/ PN/N/NI	**Y**/PY/ PN/N/NI	**Y**/PY/ PN/N/NI	**Y**/PY/ PN/N/NI	**Y**/PY/ PN/N/NI
Madani, A.S., 2012 [66]	Y/**PY**/ PN/N	**Y**/PY/ PN/N/NI	**Y**/PY/ PN/N/NI	**Y**/PY/ PN/N/NI	**Y**/PY/ PN/N/NI	**Y**/PY/ PN/N/NI	**Y**/PY/ PN/N/NI
Law, C., 2014 [67]	Y/**PY**/ PN/N	**Y**/PY/ PN/N/NI	**Y**/PY/ PN/N/NI	**Y**/PY/ PN/N/NI	**Y**/PY/ PN/N/NI	**Y**/PY/ PN/N/NI	**Y**/PY/ PN/N/NI
Lin, C., 2014 [68]	Y/**PY**/ PN/N	**Y**/PY/ PN/N/NI	**Y**/PY/ PN/N/NI	**Y**/PY/ PN/N/NI	**Y**/PY/ PN/N/NI	**Y**/PY/ PN/N/NI	**Y**/PY/ PN/N/NI
Martin-Fernandez, E., 2018 [69]	**Y**/PY/ PN/N	**Y**/PY/ PN/N/NI	**Y**/PY/ PN/N/NI	**Y**/PY/ PN/N/NI	**Y**/PY/ PN/N/NI	**Y**/PY/ PN/N/NI	**Y**/PY/ PN/N/NI
Shahriari, S., 2019 [70]	**Y**/PY/ PN/N	**Y**/PY/ PN/N/NI	**Y**/PY/ PN/N/NI	**Y**/PY/ PN/N/NI	**Y**/PY/ PN/N/NI	**Y**/PY/ PN/N/NI	**Y**/PY/ PN/N/NI
Wolf, L., 2019 [71]	Y/**PY**/ PN/N	**Y**/PY/ PN/N/NI	**Y**/PY/ PN/N/NI	**Y**/PY/ PN/N/NI	**Y**/PY/ PN/N/NI	**Y**/PY/ PN/N/NI	**Y**/PY/ PN/N/NI
Tulsani, M., 2020 [72]	Y/**PY**/ PN/N	**Y**/PY/ PN/N/NI	**Y**/PY/ PN/N/NI	**Y**/PY/ PN/N/NI	**Y**/PY/ PN/N/NI	**Y**/PY/ PN/N/NI	**Y**/PY/ PN/N/NI
Ebadian, B., 2020 [73]	Y/**PY**/ PN/N	**Y**/PY/ PN/N/NI	**Y**/PY/ PN/N/NI	**Y**/PY/ PN/N/NI	**Y**/PY/ PN/N/NI	**Y**/PY/ PN/N/NI	**Y**/PY/ PN/N/NI
Schmidt, A., 2021 [74]	Y/**PY**/ PN/N	**Y**/PY/ PN/N/NI	**Y**/PY/ PN/N/NI	**Y**/PY/ PN/N/NI	**Y**/PY/ PN/N/NI	**Y**/PY/ PN/N/NI	**Y**/PY/ PN/N/NI
Gülsoy, M., 2022 [75]	Y/**PY**/ PN/N	**Y**/PY/ PN/N/NI	**Y**/PY/ PN/N/NI	**Y**/PY/ PN/N/NI	**Y**/PY/ PN/N/NI	**Y**/PY/ PN/N/NI	**Y**/PY/ PN/N/NI
Gao, J., 2022 [76]	Y/**PY**/ PN/N	**Y**/PY/ PN/N/NI	**Y**/PY/ PN/N/NI	**Y**/PY/ PN/N/NI	**Y**/PY/ PN/N/NI	**Y**/PY/ PN/N/NI	**Y**/PY/ PN/N/NI
Risk of bias judgements	MODERATE	SERIOUS	MODERATE	LOW	MODERATE	MODERATE	MODERATE

## Data Availability

All data generated or analysed during this study are included in this article.
